# Molecular convergence of luminal androgen receptor and molecular apocrine defines a distinct entity in ER-negative breast tumors

**DOI:** 10.1186/s13058-025-02187-3

**Published:** 2025-12-27

**Authors:** Adrien Borgel, Etienne Camenen, Catherine Miquel, Philippe Bertheau, Luis Teixeira, Jacqueline Lehmann-Che

**Affiliations:** 1 Université Paris Cité, Inserm, Institut de Recherche Saint Louis (IRSL), 75010 Paris, France; 2 Molecular Oncology Unit, AP-HP, Saint Louis Hospital, 75010 Paris, France; 3 Department of Pathology, AP-HP, Saint Louis Hospital, 75010 Paris, France; 4 Breast Diseases Unit, AP-HP, Saint Louis Hospital, 75010 Paris, France

**Keywords:** Breast cancer, Luminal androgen receptor, Molecular apocrine breast cancer, Tumor heterogeneity, Diagnostic biomarkers, Gene expression profiling

## Abstract

**Background:**

Estrogen receptor–negative breast cancers are clinically heterogeneous. Two subtypes, Luminal Androgen Receptor and Molecular Apocrine Breast Cancers, are both defined by androgen signaling but identified through distinct transcriptomic classifiers. Their relationship remains unclear despite overlapping features.

**Methods:**

We analyzed transcriptomic and genomic data from public and institutional cohorts using two classification systems. Gene set enrichment and immune deconvolution analyses were performed to characterize differences between overlapping and discordant tumors. A four-gene signature was developed and validated using RNA sequencing and RT-qPCR on both fresh-frozen and formalin-fixed samples.

**Results:**

Substantial overlap was observed between the two subtypes, with shared activation of androgen and PI3K/AKT/mTOR signaling pathways. Discordant tumors were enriched in immune and stromal signals. A four-gene signature (*AR, FOXA1, SPDEF, TFF3*) identified overlapping tumors with high sensitivity and specificity across datasets and remained accurate in RT-qPCR assays on FFPE material.

**Conclusion:**

Luminal Androgen Receptor and Molecular Apocrine Breast Cancers constitute a single molecular entity within estrogen receptor–negative breast cancers. The validated four-gene signature enables robust clinical identification and may guide future therapeutic stratification.

**Supplementary Information:**

The online version contains supplementary material available at 10.1186/s13058-025-02187-3.

## Background

Breast cancer is a highly heterogeneous pathology that remains a major cause of cancer-related death in women despite numerous diagnostic and therapeutic advances. Treating triple-negative breast cancers (TNBCs) is particularly challenging due to both their lack of standard breast cancer biomarkers (ER, PR, HER2) and consequently treatment options and their molecular heterogeneity. Over the last decade, growing interest in treating TNBC has led to multiple classification proposals [1–3]. Despite differing methods, but invariably by transcriptomic analysis, these classifications consistently identify a subgroup of triple-negative tumors with luminal transcriptional characteristics that overexpress the androgen receptor (AR), known as Luminal Androgen Receptor (LAR). This subtype is now a well-defined TNBC category, and highlights potential molecular targets for LAR tumors, but which currently are understudied and lack standardized identification and treatment options. However, LARs exhibit an enrichment of HER2 and luminal PAM50 subtypes, suggesting that they should not be classified as TNBC [4].

In clinical practice, still today, breast cancer classification is largely based on the immunohistochemical status of ER, PR, and HER2. Tumors are categorized as either triple-negative or basal-like (TNBC) (ER-/PR-/HER2-), luminal (ER + /PR + /HER2-), or HER2 + (regardless of ER/PR status), a quite rough classification. In 2012, Guedj et al. proposed a six-group classification also based on transcriptomic data, named CITBCMST to create better-defined breast cancer subtypes [5]. Notably, they broke down the previous HER2 + into an ER + group and an ER-/PR-/AR + group, the latter being termed molecular apocrine breast cancers (mApo or MABC). MABCs comprise approximately two-thirds ER-/PR-/AR + /HER2 + (MABC-HER2) tumors and one-third ER-/PR-/AR + /HER2- (MABC-TN) tumors. Similar to LARs, MABCs show activation of the AR-mediated signaling pathway and its downstream targets. Although CITBCMST is less commonly used in literature compared to TNBCtype, as the latter focuses more on TNBCs that lack treatment options, it offers valuable insights into potential common targets by investigating the HER2 activation pathway. This convergence calls for a deeper investigation into the relationship between these two subtypes. This study examines whether LAR and MABC subgroups are the same entity defined by different methods and proposes an expression signature for efficient LAR/MABC tumor identification in routine practice.

## Materials and methods

### Publicly available datasets

We analyzed transcriptomic and genomic data from three publicly available breast cancer cohorts. The METABRIC dataset includes normalized microarray expression profiles of 1,981 fresh-frozen (FF) primary breast cancer samples analyzed on the Illumina HT-12 v3 array [6], among which 348 were classified as TNBC [7]. Briefly, TNBC specimens were identified using expression levels of ESR1, PGR, and ERBB2, and the available immunohistochemistry (IHC) information. Single nucleotide variants (SNVs) and copy number variations (CNVs) were obtained from the original METABRIC publication. In our analysis, only *ERBB2* amplifications were retained, and were considered distinct from gains.

The Cancer Genome Atlas (TCGA) dataset includes all 176 triple-negative breast cancer (TNBC) samples from the full TCGA cohort, based on RNA-seq data from fresh-frozen primary tumors [8].

Finally, the Fudan University Shanghai Cancer Center (FUSCC) dataset includes RNA-seq data from 330 fresh-frozen primary TNBC samples [9].

### Saint-Louis cohorts

A total of 42 fresh-frozen tumor samples from female ER- patients operated at the Breast Cancer Center of Saint-Louis Hospital between 2010 and 2021 were collected. The patients’ ages ranged from 29 to 82 years (mean = 58.8). The cohort is referred to as SLSdiscovery. RNA sequencing was performed using the Illumina NextSeq sequencer and reagents. ER, PR, AR, with > 10% cut-off, and HER2 immunohistochemistry, as well as tumor grading, were performed according to current international guidelines [10, 11]. Additionally, for 38/42 cases, corresponding formalin-fixed, paraffin-embedded (FFPE-preserved) samples were also available.

A second independent cohort, referred to as SLSvalidation, consisted of 44 FFPE-preserved ER-negative tumor samples from female patients collected from 2021 to 2024. The patients’ ages ranged from 24 to 80 years (mean = 55.0). This cohort was used as a validation set for the RT-qPCR-based gene signature developed in this study.

Samples were provided by the biological resource center after approval of the Saint Louis hospital ethical review board (Paris, France: agreement no DC 2009-929), following the Ethics and Legal national French rules for the patients’ information and consent (ANAES, HAS and INCa). All patients were informed of the study and did not oppose it, according to our Institutional Review Board recommendations.

### Bioinformatic processing pipelines

The publicly available TCGA transcriptomic data were retrieved from cBioPortal as a count table normalized to Fragments Per Kilobase Million (FPKM). Fastq files from FUSCC, SLSdiscovery, and SLSvalidation were processed using the same in-house pipeline. First, the quality of raw sequencing reads was assessed using FastQC (v0.11.9) to evaluate metrics such as sequence quality, GC content, duplication levels, and potential adapter contamination. Low-quality reads and adapter sequences were trimmed and filtered using Fastp (v0.23.1). MultiQC (v1.11) was used to summarize quality control metrics across all samples. Reads were then pseudo-aligned to the human reference transcriptome (GRCh38, release v103) using Kallisto (v0.48), which applies a de Bruijn graph-based method for transcript-level quantification. Abundance estimates were imported using Tximport (v1.22.0), and gene-level summaries were generated based on bioMart annotations (v2.42).

All Gene Set Pathway Enrichment (GSEA) analyses were performed using the GSEA software application developed by the Broad Institute (v4.3.3), with the Hallmark gene sets from the Molecular Signatures Database (MSigDB). Transcriptomic data deconvolution was performed using MCPcounter (https://github.com/ebecht/MCPcounter/, v1.2.0).

Random forest analysis was performed using randomForest R package (v4.7-1.2). All statistical analyses were performed using R software. Comparisons of proportions were carried out using Chi-squared tests, while continuous variables were analyzed using Student’s t-tests. A *p*-value < 0.05 was considered statistically significant.

### Breast cancer subtyping

METABRIC tumor samples were classified using the CITBCMST R package (v1.0.4) [5], as this package is specifically adapted to microarray data. The classifier is based on a distance-to-centroid approach for lumA, lumB, lumC, mApo (MABC), basL, and normL subtypes. For the TNBCtype classifier, we used previously published classifications of 348 METABRIC TNBC tumor samples by Lehmann BD et al. [7]. Each TNBC tumor was classified into four TNBC subtypes (BL1, BL2, LAR, and M) or remained unclassified (UNS) using the TNBCtype web-based tool (http://cbc.mc.vanderbilt.edu/tnbc/) [4, 12].

The 42 tumor samples from the SLSdiscovery cohort were classified using the RNABC R package (https://bitbucket.org/cbligaard/rnabc/) [13], an RNAseq-adapted version of the CITBCMST classifier. We also used the TNBCtype classifier [4]. Each tumor was initially assigned a TNBCtype-6 subtype, which was subsequently converted to the TNBCtype-4 classification by retaining the subtype (BL1, BL2, M, or LAR) with the highest coefficient. The same approach was applied to the 44 samples from the SLSvalidation cohort.

For the TCGA cohort, subtyping was performed using the RNABC classifier on RNA-seq data. As with METABRIC, TNBC subtypes were retrieved from Lehmann BD et al. published subtyping [7].

### RT-qPCR Signature for FFPE samples

Thirty-eight out of the 42 tumor samples from the SLSdiscovery cohort, which were also available as FFPE material, along with all 44 samples from the SLSvalidation cohort, were analyzed by RT-qPCR using specific TaqMan^®^ gene expression assays (ThermoFisher) to assess expression levels of *AR* (Hs00171172_m1), *FOXA1 *(Hs00270129_m1), *SPDEF* (Hs01026048_m1), and *TFF3* (Hs00173625_m1) using the 2^-ΔCT^ method [13], with *RPL37A* (Hs06050959_g1) as the reference gene. Reverse transcription was performed on RNA extracts using SuperScript™ II Reverse Transcriptase (Invitrogen) and random hexamers, and consisted of a 3-min denaturation step at 65 °C, followed by primer annealing at 25 °C for 10 min, and extension at 42 °C for 1 h. After RT, cDNA samples are conserved at − 20 °C. Amplifications were performed in duplicate on a QS5 system (ThermoFisher) with a cDNA matrix (4 ng/µL). The protocol included initial activation at 95 °C for 10 min, followed by 45 cycles (15 s at 95 °C and 1 min at 65 °C). Analysis was done on QuantStudio 5 Dx. For each TaqMan^®^ gene expression assay, PCR efficacy was evaluated on a 7 points standard curve. All PCR efficiencies are between 1.01 and 1.07 and slopes are equivalent (Supplementary Fig. 1). Samples were classified as MABC/LAR if ≥ 3 out of 4 targets exceeded these cut-offs: *AR* > 5.0, *FOXA1* > 24, *SPDEF* > 3, and *TFF3* > 1.0. These cut-offs were determined using the easyROC R package (v1.3.1) on the SLSdiscovery RT-qPCR dataset to accurately identify MABC/LAR tumors defined by RNABC/TNBCtype on corresponding RNA-seq data (Supplementary Fig. 2A). The cut-offs were then validated using the SLSvalidation RT-qPCR dataset with the same process (Supplementary Fig. 2B). Comparisons of proportions were carried out using Chi-squared tests. A *p*-value < 0.05 was considered statistically significant.

## Results

### Discrepancies between TNBCtype and CITBCMST classifications highlight intra-subtype heterogeneity of MABC and LAR tumors

**Fig. 1:**
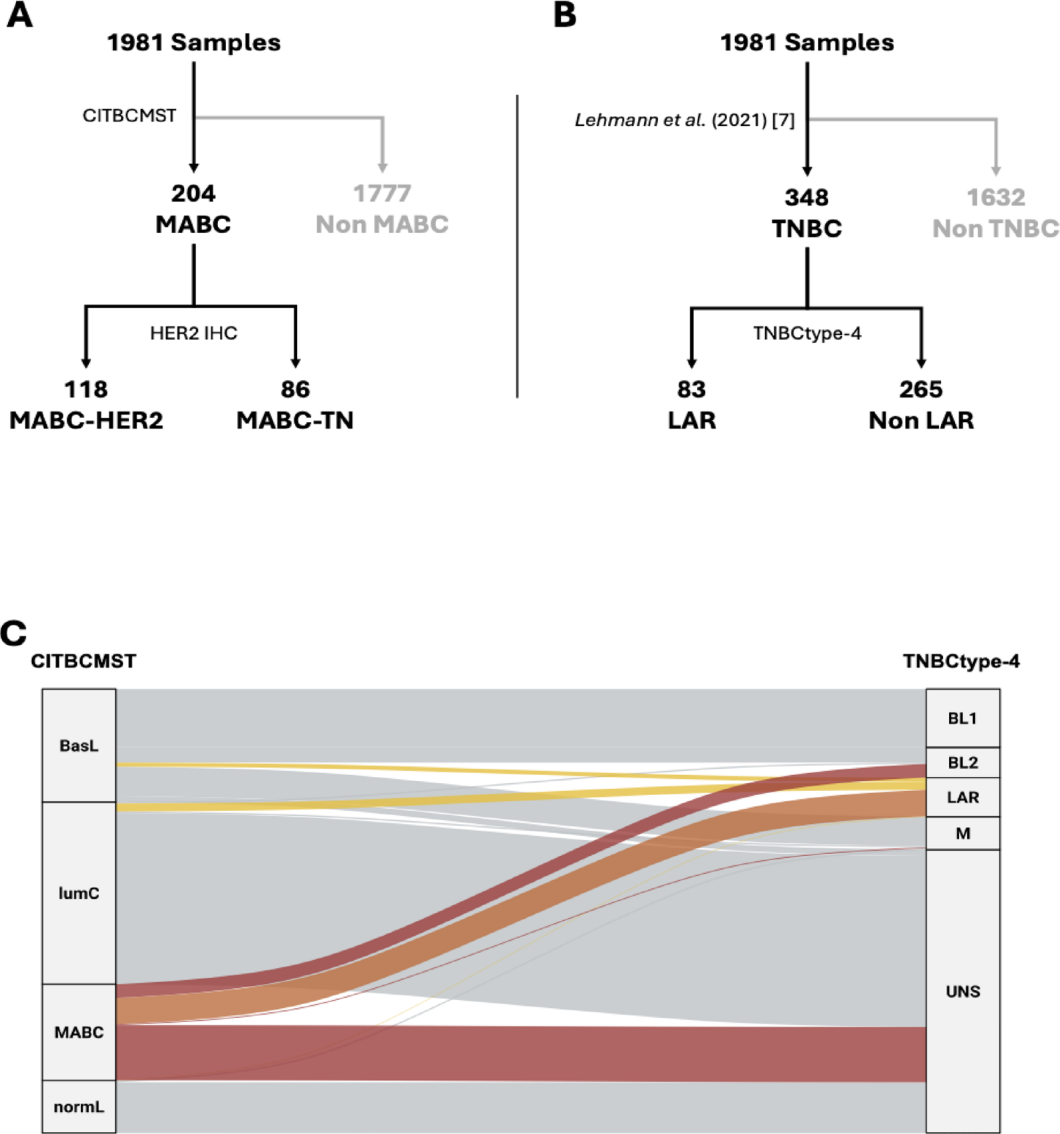
Classification of METABRIC tumors using CITBCMST and TNBCtype-4. **A** Among the 1981 samples, 204 were classified as MABC by CITBCMST; based on HER2 IHC, 118 were HER2-positive (MABC-HER2) and 86 were triple-negative (MABC-TN). LumA and lumB subgroups were excluded. **B** Independently, 348 tumors were identified as TNBC, including 83 classified as LAR using the TNBCtype-4 classifier. **C** Distribution of samples classified as MABC by the CITBCMST classifier (left) and as LAR by the TNBCtype-4 classifier (right) across the subgroups defined by the two transcriptomic signatures. Three overlapping subgroups emerge: MABC-LAR (orange), representing tumors classified as both MABC and LAR; LAR-nonMABC (yellow), corresponding to LAR tumors not classified as MABC; and MABC-nonLAR (red), corresponding to MABC tumors not classified as LAR. BasL, Basal-like; LumC, Luminal C; MABC, Molecular Apocrine Breast Cancer; NormL, Normal-like; BL1, Basal-like 1; BL2, Basal-like 2; LAR, Luminal Androgen Receptor; M, Mesenchymal; UNS, Unclassified

To investigate LARs and MABCs, we used the METABRIC cohort, which provides access to microarray-based gene expression data along with detailed pathological and clinical annotations. Analysis of METABRIC microarray data identified 83 LARs (23.9%) within the 348 TNBCs using the TNBCtype-4 classifier, and 204 MABCs in the entire cohort (n = 1981) using the CITBCMST classification (Fig. 1A and B). Based on HER2 expression determined by IHC, MABCs can be divided as 86/204 (42.2%) MABC-TN and 118/204 (57.8%) MABC-HER2. Based on our initial hypothesis, we anticipated significant overlap between the 83 LARs and 86 MABC-TN. Surprisingly, only 67.5% (56/83) of LARs were also classified as MABC-TN (Fig. 1C). Notably, 17 (20.5%) of LARs were classified as lumC, 9 (10.8%) as basL, and 1 (1.2%) as normL by CITBCMST (Fig. 1A). The high number of LAR tumors classified as lumC is intriguing, given that the lumC subtype, as described by Guedj et al., is typically characterized by ER expression, albeit lower than that of lumB, and by HER2 overexpression in approximately 40% of cases [5]. Notably, the 17 LARs classified as lumC showed a significantly lower *ESR1* expression compared to the rest of lumC tumors (*p*-value < 0,001). The fact that one fifth of LARs are classified as lumC by CITBCMST can be explained by the enrichment of ER-related pathways observed in LAR tumors [2, 3]. Additionally, it is well known that AR can activate numbers of estrogen responsive genes [14]. Therefore, we hypothesize that a LAR tumor could be classified as LumC if it shows a more important estrogen-related pathway activation than tumors classified as MABCs. Conversely, among the 86 MABC-TN, distribution across TNBCtype-4 subtypes was 65,1% (56/86) LARs, 33.7% (29/86) BL2, 1.2% (1/86) M and no BL1 (Fig. 1A). Notably, in 23 out of 30 MABC-nonLAR tumors, the LAR centroid had the second highest correlation coefficient. Those observations suggest a transcriptional continuum between MABC and lumC, and between LAR and BL2 subtypes, reflecting the complexity and gradual nature of luminal features within ER-negative breast cancers.

### Frequent PI3K/AKT/mTOR pathway activation and ERBB2 involvement in MABC and LAR subtypes

Approximately 30–40% of breast cancers exhibit activating *PIK3CA* mutations, leading to activation of the PI3K/AKT/mTOR signaling pathway [15]. This pathway can also be activated by mutation or amplification of RTKs, notably *ERBB2* (HER2). We investigated oncogenic mutations and amplifications in *PIK3CA* and *AKT1*, and loss-of-function mutations or deletions in *PTEN*, as well as *ERBB2* amplification, all of which lead to the activation of the PI3K/AKT/mTOR pathway. We limited our analysis to alterations reported as pathogenic by OncoKB [16], in the MABC and LAR subtypes from the METABRIC cohort.

**Fig. 2:**
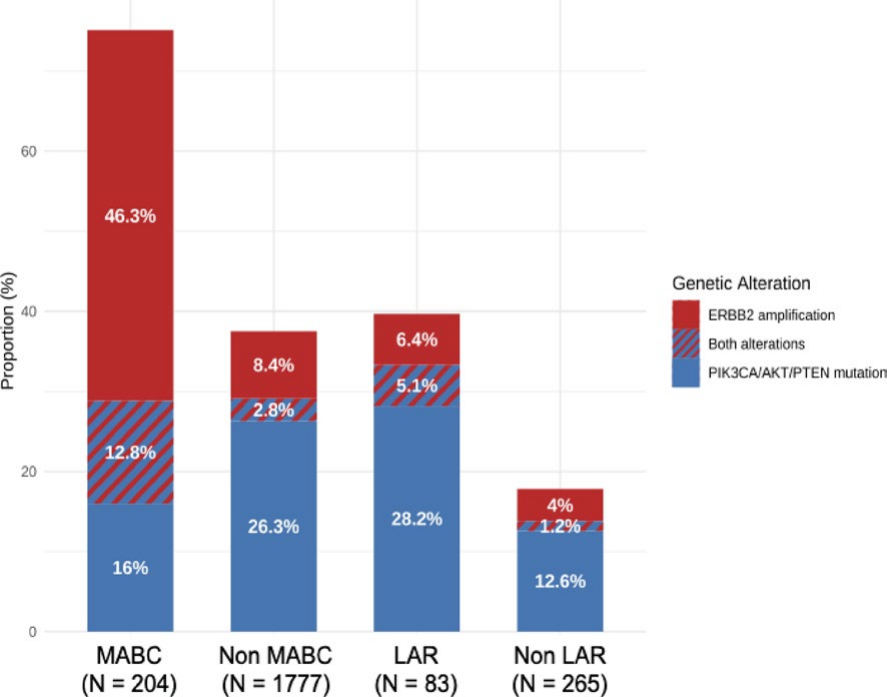
Distribution of *ERBB2* amplification (red) and *PIK3CA/AKT1/PTEN* mutations (blue) across breast cancer subtypes in the METABRIC cohort. Subgroups are defined based on either MABC or LAR classification. Genetic alterations are mutually exclusive or co-occurring, with co-alterations represented by striped bars

MABCs had no differences in the frequency of alterations on the *PIK3CA, AKT1 and PTEN* genes (28.8%) compared to the rest of the entire METABRIC cohort (29.1%, *p*-value = 0.127), due to the large proportion of ER + luminal samples, classically frequently PIK3CA mutated (Fig. 2). In contrast, MABCs showed a greater alteration rate when compared to BasL tumors (28.7% vs. 13.1%, *p*-value < 0.01). Additionally, *ERBB2* amplifications were significantly more frequent in MABCs compared to non-MABCs (59.1% vs. 11.2%, *p*-value < 0.001). This enrichment was primarily attributable to the MABC-HER2 subclass, as 95.0% (112/118) of them have an amplification of *ERBB2*. Overall, MABCs exhibited significantly more PI3K/AKT/mTOR pathway activation (75.1%) compared to non-MABCs (37.5%).

LAR tumors similarly exhibited significantly more frequent *PIK3CA/AKT1/PTEN* gene alterations (33.4% vs. 13.8%) and *ERBB2* amplifications (11.5% vs. 5.2%) compared to other TNBCs, resulting in overall higher activation (39.7% vs. 17.8%) (Fig. 2). LARs also had higher *ERBB2* mRNA expression levels compared to BL1, BL2, and M subtypes (*p*-value < 0.001). Notably, 8/79 (10.1%) of LARs showed *ERBB2* amplification, despite being classified as TNBC. Altogether, the frequent activation of the PI3K/AKT/mTOR pathway and the involvement of *ERBB2* in both MABC and LAR tumors further support their molecular overlap and reinforce the rationale for considering them as a unified subgroup within ER-negative breast cancers.

### Transcriptomic profiling highlights biological divergence among discordant LAR and MABC tumors

We performed a Gene Set Enrichment Analysis (GSEA) comparing MABCs to BasL tumors from the METABRIC cohort. As expected, the MABC group showed enrichment in pathways related to androgen metabolism and broader steroid metabolism. Interestingly, MABCs also displayed enrichment in the estrogen early response pathway, consistent with the significant overlap of downstream targets between AR and ER [14]. Additionally, we observed enrichment of the PI3K/AKT/mTOR pathway, aligning with our MABC mutational analysis. Similar enrichment in AR-related pathways was found when comparing LAR to non-LAR TNBCs.

**Fig. 3:**
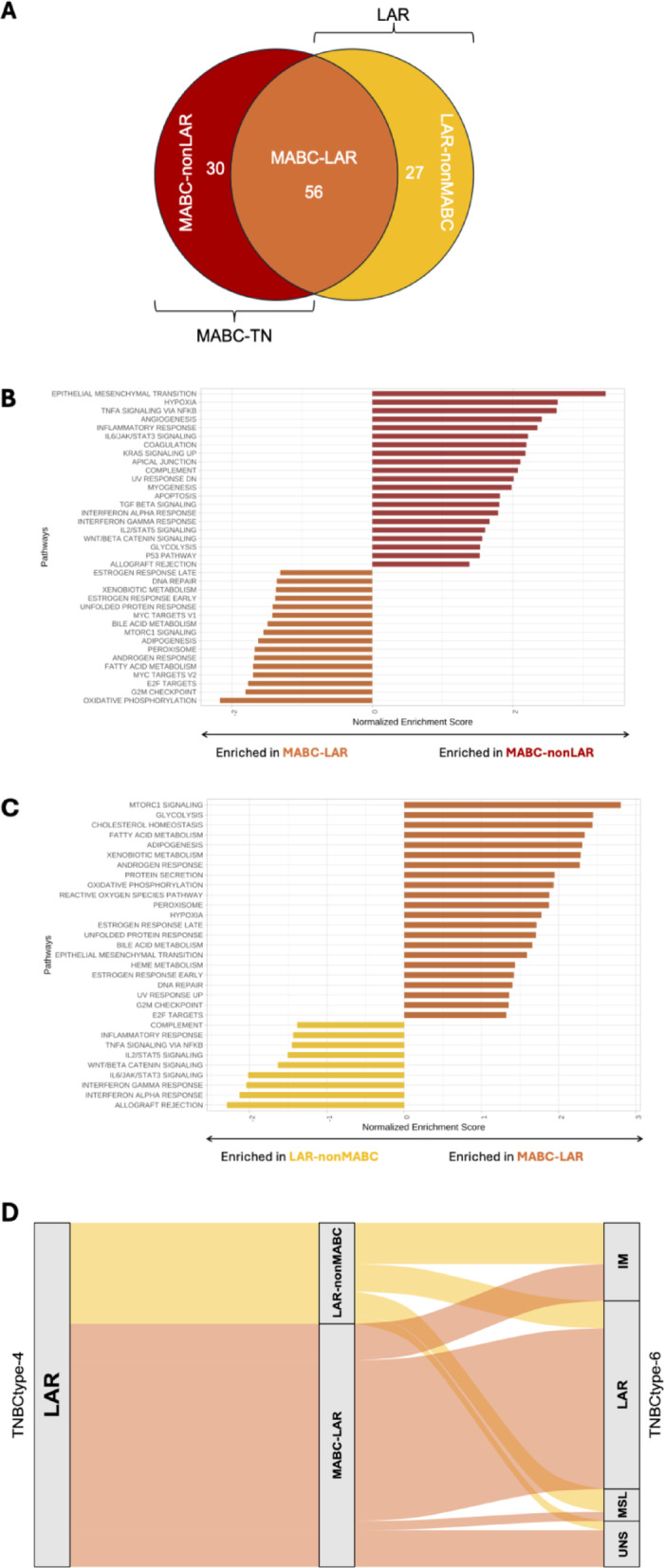
**A** Venn diagram illustrating the distribution of Triple negative MABC (MABC-TN) and LAR tumors among triple-negative breast cancers (TNBC) in the METABRIC cohort. Only TNBC cases are shown; HER2-positive MABC tumors were deliberately excluded. Three subgroups are highlighted: MABC-LAR (orange), LAR-nonMABC (yellow) and MABC-nonLAR (red). **B** Hallmark Gene Set Pathway Enrichment (GSEA) analysis between MABC-LAR and either MABC-nonLAR **C** or LAR-nonMABC. **D** Sankey diagram illustrating the distribution of LAR tumors (as defined by the TNBCtype-4 classifier, left) into MABC-LAR (orange) and LAR-nonMABC (yellow) subgroups, and their subsequent classification by the original TNBCtype-6 model (right). IM, Immunomodulatory, LAR, Luminal Androgen Receptor, MSL, Mesenchymal Stem-Like, UNS, unclassified

To further understand the high number of discordant samples between LARs and MABCs, we examined activated pathways within three subgroups: MABC-LAR, MABC-nonLAR and LAR-nonMABC (Fig. 3A). Compared to the discordant subgroups, MABC-LAR tumors showed predominant enrichment in androgen and steroid metabolism pathways, consistent with the MABC vs BasL comparison (Fig. 3B and C). In contrast, MABC-nonLAR tumors exhibited enrichment in 21 pathways, including several related to immune response (n = 9) and epithelial-mesenchymal transition (EMT). Similarly, the 10 pathways significantly enriched in LAR-nonMABC compared to MABC-LAR were all immune-related. These results highlight distinct biological profiles among discordant tumors, with MABC-nonLAR combining features of immune activation and EMT, and LAR-nonMABC characterized predominantly by immune-related features, while MABC-LAR tumors remain more specifically associated with steroid-related signaling.

### Immune and stromal infiltration shapes transcriptomic identity of discordant tumors

Overall, the pathway enrichment analysis shows an enrichment of AR related pathways in the core MABC-LAR subgroup and an enrichment of immune related pathways in both outlier subgroups. We hypothesize that these differences are due to sample heterogeneity rather than a biological signal. Firstly, these are microarray data, which do not allow for the extraction of signals from specific cell types. Additionally, there is no information on the type of surgery (microbiopsy vs. lumpectomy) from which the METABRIC cohort samples were derived [6]. However, semiquantitative cellularity data were available. MABC-LAR samples were significantly enriched in highly cellular samples (> 70%) compared to MABC-nonLAR (53.6% vs. 27.3%, *p*-value < 0.01), but not when compared to LAR-nonMABC.

To further investigate the biological basis underlying the transcriptomic divergence between discordant MABC and LAR tumors, we performed cellular deconvolution to assess differences in immune and stromal composition. We found a significantly higher proportion of fibroblasts in the MABC-nonLAR subgroup compared to MABC-LAR (*p*-value < 0.001), consistent with both the observed differences in sample cellularity and the enrichment of the EMT pathway. This association is in line with previous findings showing that EMT-related gene expression signatures in bulk tumor data often reflect stromal, and especially fibroblast, abundance rather than a true EMT in cancer cells [17, 18]. These differences are consistent with our GSEA results.

To characterize the molecular features of MABC-LAR and LAR-nonMABC tumors, we applied the TNBCtype classification developed by Lehmann et al. While the original version published in 2011 defined six subtypes (TNBCtype-6: BL1, BL2, M, LAR, IM, and MSL), a revised version was proposed in 2016 (TNBCtype-4), which excludes the Immunomodulatory (IM) and Mesenchymal Stem-Like (MSL) subtypes. These two groups were removed after it was shown that their gene expression profiles largely reflected signals from infiltrating immune cells and stromal components rather than tumor-intrinsic features. In the TNBCtype-4 classifier, IM and MSL samples are reassigned to their second most correlated intrinsic subtype. In our analysis, 66.0% (35/53) of MABC-LAR samples were classified as LAR by the original TNBCtype-6 classifier, compared to only 22.2% (6/27) of LAR-nonMABC (Fig. 3D). The remaining MABC-LAR samples were classified as IM (15.1%, 8/53), MSL (3.8%, 2/53), or UNS (15.1%, 8/53). Among the LAR-nonMABC group, a larger fraction was assigned to non-intrinsic subtypes: 33.3% (9/27) to IM, 18.5% (5/27) to MSL, and 7.4% (2/27) to UNS. These results indicate that the transcriptomic profiles of LAR-nonMABC tumors are more influenced by immune and stromal components than those of MABC-LAR, consistent with our GSEA findings.

### Identification of a 4-gene signature distinguishing LAR/MABC tumors using RNA-seq data

Based on the preceding analyses, LAR and MABC tumors appear to represent overlapping biological entities. Given their shared transcriptomic and genomic characteristics, we hypothesized that a common gene expression signature could be used to identify these tumors within the broader group of ER-negative breast cancers.

Using RNAseq data from the TCGA, FUSCC and SLSdiscovery cohorts, we selected four genes that are highly overexpressed in the MABC and LAR populations compared to TNBC and are recognized as AR targets [5, 19, 20]: *AR, FOXA1, SPDEF* and *TFF3*. Those four genes are recurrently identified in this subtype [1, 5, 20, 21]. Beyond their strong expression, these four genes play central roles in the oncogenesis of molecular apocrine breast cancers [22–27]. To validate these genes, we performed a random forest analysis using their RNA-seq expression levels, normalized to *TBP* (a housekeeping gene), to assess whether the expression of these four genes alone could effectively classify LAR/MABC tumors, within a population of ER- breast cancers. The model was trained on the TCGA cohort and subsequently validated on both the FUSCC and SLSdiscovery cohorts. The model was able to identify LAR and MABC tumors using RNA-seq data with high accuracy. Against the TNBCtype-4 signature, it achieved a sensitivity and a specificity of 98.5% and 83.1% respectively, in the FUSCC cohort, and 100% and 76% in the SLSdiscovery cohort. When compared to the RNABC signature, sensitivity and specificity were 96.8% and 83.1% in FUSCC, and 100% and 90.5% in SLSdiscovery, respectively. These results support the relevance of the four-gene signature for detecting LAR/MABC tumors.

### Clinical implementation of the signature using RT-qPCR on FFPE samples

However, as RNA sequencing is not routinely used in clinical practice for breast cancer, we next sought to determine whether this signature could be applied using RT-qPCR on FFPE tissue, a format more compatible with clinical workflows. Importantly, although LAR tumors are always triple-negative, MABCs can be either HER2-negative or HER2-positive, and thus not always part of the TNBC group. Therefore, we evaluated the signature across the broader group of ER-negative tumors, which includes both LARs and MABCs.

First, we utilized the 2^−∆CT^ method with *RPL37A* as the housekeeping gene to test these four genes on 38 FFPE preserved samples from the SLSdiscovery cohort, after proper PCR efficacy validation for each gene (Supplementary Fig. 1). *RPL37A* was selected over *TBP*, which was used for normalization in the RNA-seq analysis, due to its previous validation in FFPE-preserved material [28]. The RT-qPCR signature correctly classified 17/18 MABCs and 18/20 non-MABCs, demonstrating sensitivity and specificity of 94.4% and 90.0%, respectively, compared to the gold standard RNABC classifier (Table 1A). When referenced against the TNBCtype-4 signature, the RT-qPCR signature correctly classified 15/16 LARs and 18/22 non-LARs, with a sensitivity of 93.8% and specificity of 81.1% (Table 1A). These findings indicate no significant difference between the transcriptome-derived RNABC or TNBCtype-4 classifications and the RT-qPCR 4-gene signature (*p*-value < 0.05). This signature can be reliably used on FFPE-preserved samples, extending its utility in clinical settings.

**Table 1:**
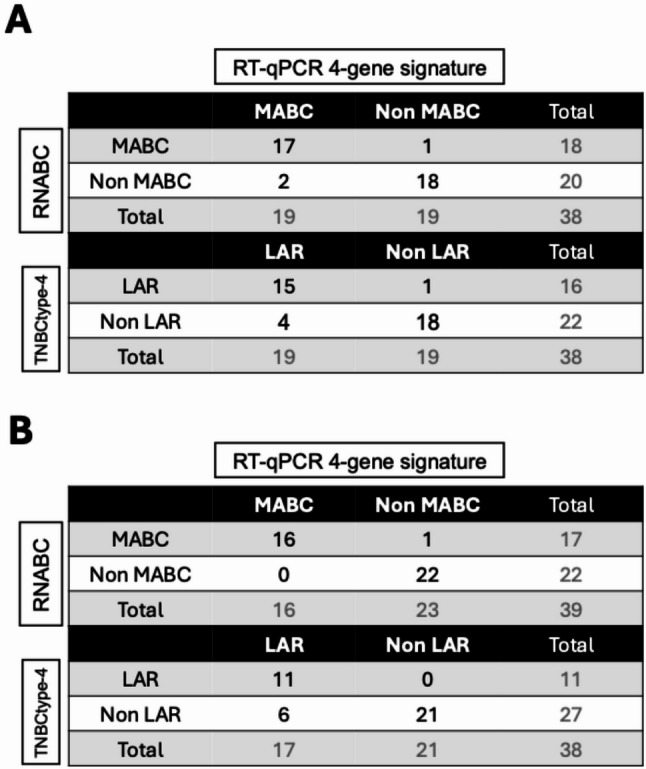
Contingency table comparing the 4-gene RT-qPCR signature with the gold standard classifiers TNBCtype-4 and RNABC (CITBCMST adapted to RNAseq data) in the (A) SLSdiscovery and (B) SLSvalidation cohorts. Samples that could not be classified by the gold standards were excluded from the analysis

To confirm the robustness of our RT-qPCR 4-gene signature, we validated its performance on the independent SLSvalidation cohort of 44 ER-negative tumors. This cohort was firstly analyzed by RNA sequencing, allowing classification with both RNABC and TNBCtype-4. Using RNABC, we identified 17 MABC, 22 non-MABC, and 5 normL tumors (Table 1B). TNBCtype classification identified 11 LAR, 27 non-LAR, and 6 unclassifiable tumors. This cohort was then analyzed using the same RT-qPCR protocol and classification thresholds established in the SLSdiscovery cohort. The signature achieved a sensitivity of 94.1% and a specificity of 100% using RNABC as reference, and 100% and 77.8% respectively using TNBCtype-4, further supporting its reproducibility and potential for clinical use in FFPE-preserved samples (*p*-value < 0.05).

To better understand the discrepancies between classifiers, we reviewed the discordant samples (Supplementary Table 1). Most corresponded to tumors with strong immune or stromal components. Others displayed hybrid transcriptional features bridging MABC/LAR and Basal-like programs, suggesting biological heterogeneity rather than technical misclassification.

## Discussion

Recent clinical trials have underscored the importance of accurate subtyping in TNBC to guide therapeutic decisions [29, 30]. The LARs and MABCs subtypes have attracted growing attention due to their shared molecular features and potential sensitivity to anti-androgen therapies. However, these two entities are still inconsistently defined, and their boundaries remain blurred.

In this study, we addressed this issue by demonstrating that LAR and MABC largely overlap at the molecular level and likely represent a unified biological entity within ER-negative breast cancers. Through comparative analysis of two independent classification systems (TNBCtype and CITBCMST/RNABC), we identified a core group of LAR/MABC tumors with highly concordant profiles, as well as peripheral subgroups displaying intermediate features. These findings suggest that apparent subtype discrepancies often reflect transcriptional gradients or intratumoral heterogeneity rather than fundamental molecular divergence.

The inconsistencies between classifiers also highlight the limitations of rigid subtype assignments. Similar concerns led Lehmann et al. to revise the original TNBCtype-6 classifier by excluding the IM and MSL subtypes, which were driven by immune and stromal components rather than intrinsic tumor biology [4]. In line with this, we observed that discordant tumors, LAR-nonMABC and MABC-nonLAR, were enriched in immune signalling pathways and stromal cell population, and often classified as IM or MSL when using the original version of the TNBCtype classifier.

In contrast to these differences in classification, MABC and LAR tumors share highly similar molecular characteristics at the genetic and signaling levels. Both subtypes show frequent alterations in the PI3K/AKT/mTOR pathway, and a significant subset of tumors display *ERBB2* amplification or overexpression, despite being triple-negative by standard IHC. These shared features provide strong biological rationale for considering LAR/MABC as a single molecular entity within ER-negative breast cancers.

To support this hypothesis, we developed a 4-gene signature based on the expression of *AR, FOXA1, SPDEF,* and *TFF3 genes*, all known *AR* targets [19]. This signature proved robust across multiple datasets and platforms: it accurately identified LAR/MABC tumors using microarray data (METABRIC), RNA-seq data from fresh-frozen tumors (TCGA, FUSCC, SLSdiscovery), and even RNA-seq data from FFPE samples (SLSvalidation).

Importantly, it was successfully transferred into a RT-qPCR assay applicable to clinical settings. In both discovery and independent validation cohorts, the assay achieved high sensitivity and specificity, demonstrating not only its technical robustness but also its feasibility in real-world conditions.

While AR IHC remains the most widely used method to screen for LAR tumors due to its simplicity and accessibility, it suffers from a lack of standardization. No consensus exists regarding the antibody clone to use, and positivity thresholds vary across studies and countries, limiting its reproducibility and diagnostic accuracy. This variability likely contributes to the poor predictive value of AR IHC alone, as highlighted by the modest outcomes of clinical trials evaluating anti-AR therapies in AR-positive TNBCs [31–35]. The 4-gene RT-qPCR signature we propose offers a standardized, easy-to-implement alternative for patient stratification, with potential application as an inclusion criterion in future clinical trials targeting the LAR/MABC axis.

Finally, our findings invite a re-evaluation of how LAR tumors are classified. While they are currently considered a subset of TNBC, their frequent overlap with MABC tumors, including HER2-positive cases, suggests that LAR should be more broadly defined as a subset of ER-negative breast cancers. This revised framing better aligns with their molecular profile and has important implications for classification and therapeutic stratification. In particular, the frequent activation of the PI3K/AKT/mTOR pathway in these tumors highlights additional therapeutic opportunities beyond androgen blockade. Notably, recent preclinical studies have shown that enzalutamide-resistant LAR models harboring *PIK3CA* or *AKT1* mutations can be effectively targeted by PI3K and mTOR inhibitors, while showing limited or no response to AR inhibition [36]. These findings highlight the potential clinical utility of PI3K/AKT/mTOR-targeted therapies in this subgroup. Combinatorial strategies may remain of interest, but PI3K pathway inhibition, whether as monotherapy in selected cases or in combination with other agents, may represent a promising approach that warrants further clinical evaluation.

## Supplementary Information

Below is the link to the electronic supplementary material.

Supplementary Material 1.

Supplementary Material 2.

## Data Availability

No datasets were generated or analysed during the current study.

## Abbreviations

AR: Androgen receptor
BL1: Basal-like 1
BL2: Basal-like 2
CNV: Copy number variation
EMT: Epithelial-mesenchymal transition
ER: Estrogen receptor
FFPE: Formalin-fixed, paraffin-embedded
FPKM: Fragments per kilobase million
FUSCC: Fudan University Shanghai Cancer Center
GSEA: Gene set enrichment analysis
HER2: Human epidermal growth factor receptor 2
IHC: Immunohistochemistry
IM: Immunomodulatory
LAR: Luminal androgen receptor
MABC: Molecular apocrine breast cancer
M: Mesenchymal
MSL: Mesenchymal stem-like
PR: Progesterone receptor
qPCR: Quantitative polymerase chain reaction
RT-qPCR: Reverse transcription quantitative PCR
SNV: Single nucleotide variant
TNBC: Triple-negative breast cancer
UNS: Unclassified
