# Supplementary material for: Molecular convergence of luminal androgen receptor and molecular apocrine defines a distinct entity in ER-negative breast tumors
Source: Breast Cancer Res. 2025 Dec 27;28:26. doi: 10.1186/s13058-025-02187-3 (PMC12853821; doi:10.1186/s13058-025-02187-3)
Supplement: Supplementary file 2 — Supplementary Material 2. [file 13058_2025_2187_MOESM2_ESM.pdf]

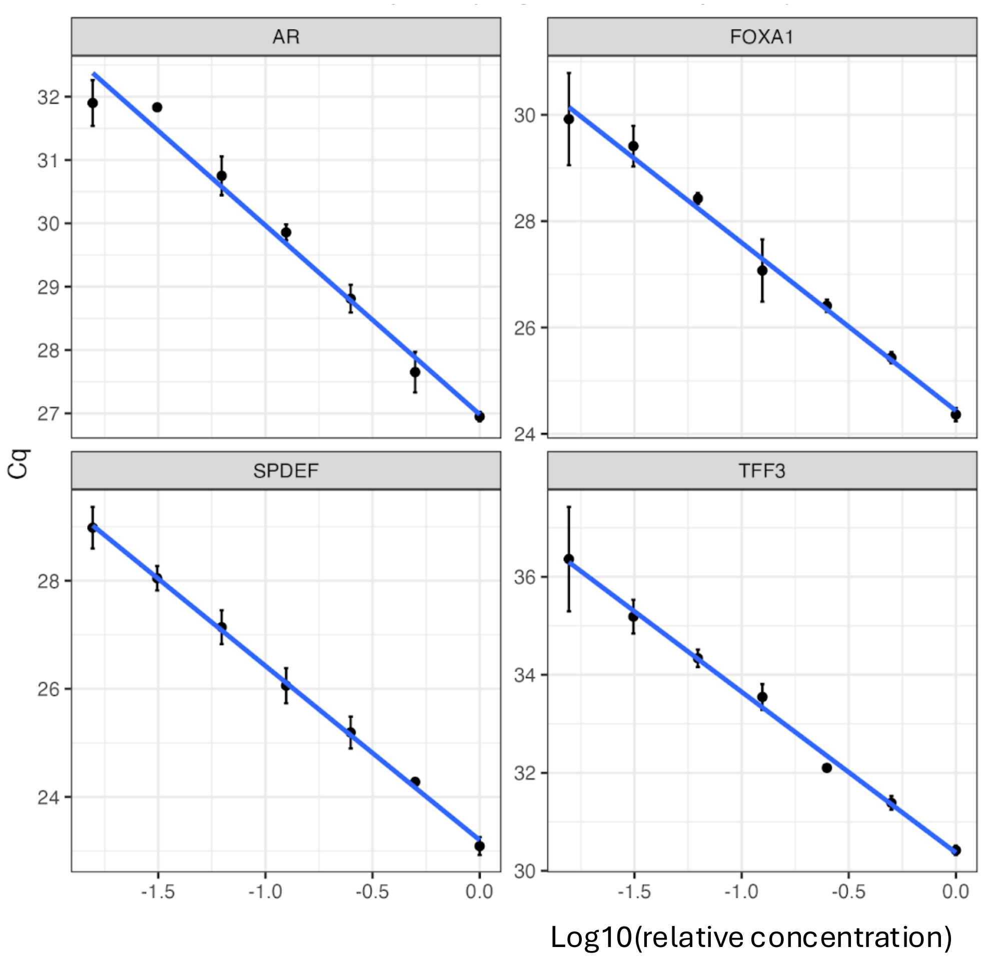

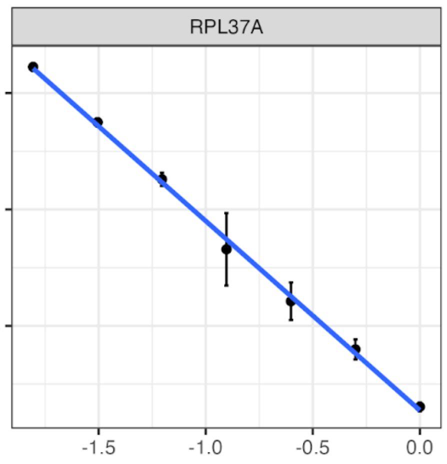

Target	Slope	Intercept	R^2	Efficiency fraction	Efficiency percentage	N points	Within QC
AR	-3.298	26.826	0.997	1.010	100.992	7	OK
FOXA1	-3.160	24.437	0.992	1.073	107.229	7	OK
RPL37A	-3.253	24.550	0.998	1.030	102.980	7	OK
SPDEF	-3.222	23.203	0.999	1.044	104.352	7	OK
TFF3	-3.281	30.371	0.995	1.017	101.717	7	OK

Supplementary Figure 1. RT-qPCR standard curves for the 4-gene signature and reference gene.
Standard curves were generated for *AR*, *FOXA1*, *SPDEF*, *TFF3*, and the housekeeping gene *RPL37A* using serial dilutions of cDNA. Each point represents the mean quantification cycle (Cq) ± standard deviation from technical replicates. The linear regression line (in blue) shows the expected inverse relationship between Cq values and the logarithm of relative cDNA concentration (log_{10} scale), confirming high amplification efficiency and assay reproducibility for all targets.

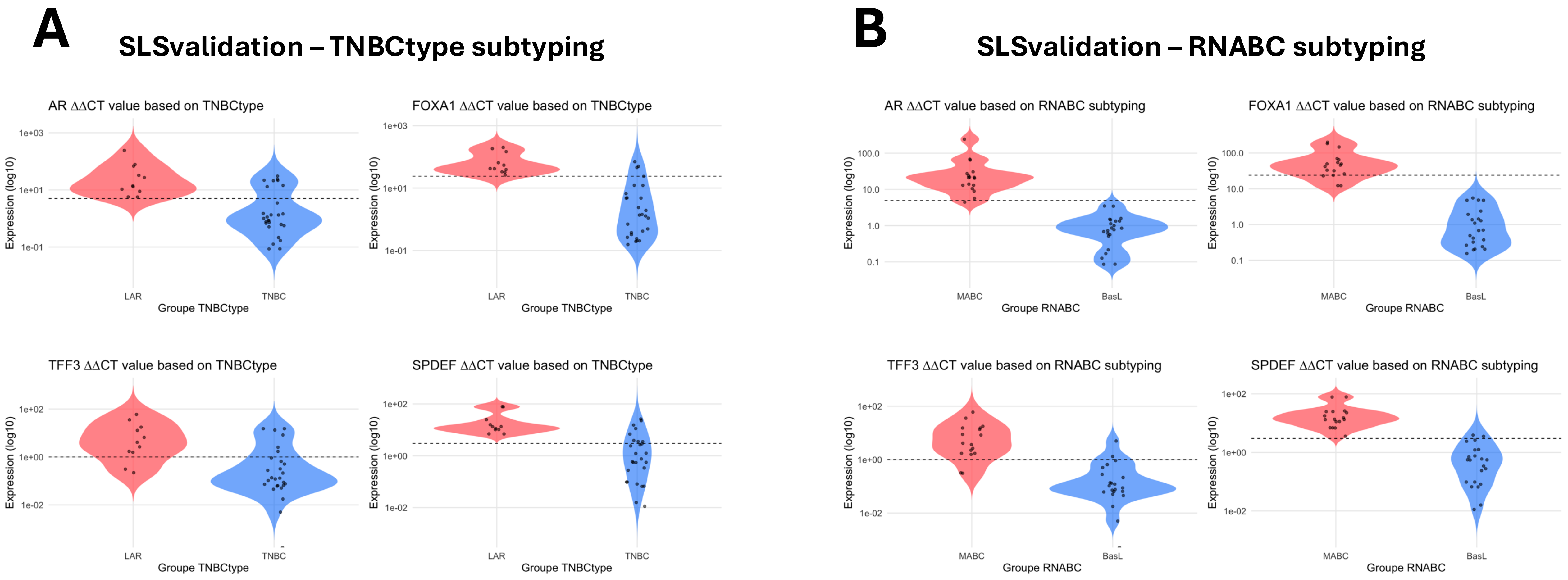

Supplementary Figure 2. Validation of the 4-gene expression signature in the SLSvalidation cohort using two independent molecular classifiers.
(A) Violin plots showing the ΔΔCT values (log10 scale) of *AR*, *FOXA1*, *SPDEF*, and *TFF3* in tumors classified as LAR or TNBC according to the TNBCtype-4 classifier. **(B)** Corresponding expression patterns in tumors classified as MABC or BasL according to the RNABC classifier.
For each gene, red indicates LAR/MABC tumors and blue indicates TNBC/BasL tumors. The dashed lines represent the median threshold used to distinguish high and low expression.
